# Ethnoagroforestry: integration of biocultural diversity for food sovereignty in Mexico

**DOI:** 10.1186/s13002-016-0127-6

**Published:** 2016-11-23

**Authors:** Ana Isabel Moreno-Calles, Alejandro Casas, Alexis Daniela Rivero-Romero, Yessica Angélica Romero-Bautista, Selene Rangel-Landa, Roberto Alexander Fisher-Ortíz, Fernando Alvarado-Ramos, Mariana Vallejo-Ramos, Dídac Santos-Fita

**Affiliations:** 1 Escuela Nacional de Estudios Superiores Unidad Morelia (ENES), Universidad Nacional Autónoma de México. UNAM, Campus Morelia, Antigua Carretera a Pátzcuaro No. 8701, Col. Ex-Hacienda de San José de la Huerta, Morelia, 58190 Michoacán México; 2 Instituto de Investigaciones en Ecosistemas y Sustentabilidad (IIES), Universidad Nacional Autónoma de México. UNAM, Campus Morelia, Antigua Carretera a Pátzcuaro No. 8701, Col. Ex-Hacienda de San José de la Huerta, Morelia, 58190 Michoacán México; 3 Centro de Investigaciones en Geografía Ambiental, Universidad Nacional Autónoma de México (CIGA). UNAM, Campus Morelia, Antigua Carretera a Pátzcuaro No. 8701, Col. Ex-Hacienda de San José de la Huerta, Morelia, 58190 Michoacán México; 4 Centro Universitario de la Costa Sur (CUC Sur), Universidad de Guadalajara. Avenida Independencia Nacional No. 151, Colonia Centro, Autlán de Navarro, 48900 Jalisco México; 5 Centro de Investigación en Ciencias Biológicas Aplicadas (CICBA), Universidad Autónoma del Estado de México. Instituto literario No 100, Colonia Centro, Toluca, 50000 Estado de México México

**Keywords:** Agroforestry systems, Biodiversity management, Local food systems, Small farm agriculture, Traditional agriculture

## Abstract

**Background:**

Documenting the spectrum of ecosystem management, the roles of forestry and agricultural biodiversity, TEK, and human culture for food sovereignty, are all priority challenges for contemporary science and society. Ethnoagroforestry is a research approach that provides a theoretical framework integrating socio-ecological disciplines and TEK. We analyze in this study general types of Agroforestry Systems of México, in which peasants, small agriculturalist, and indigenous people are the main drivers of AFS and planning of landscape diversity use. We analyzed the actual and potential contribution of ethnoagroforestry for maintaining diversity of wild and domesticated plants and animals, ecosystems, and landscapes, hypothesizing that ethnoagroforestry management forms may be the basis for food sufficiency and sovereignty in Mexican communities, regions and the whole nation.

**Methods:**

We conducted research and systematization of information on Mexican AFS, traditional agriculture, and topics related to food sovereignty from August 2011 to May 2015. We constructed the database *Ethnoagroforestry* based on information from our own studies, other databases, Mexican and international specialized journals in agroforestry and ethnoecology, catalogues and libraries of universities and research centers, online information, and unpublished theses. We analyzed through descriptive statistical approaches information on agroforestry systems of México including 148 reports on use of plants and 44 reports on use of animals.

**Results:**

Maize, beans, squashes and chili peppers are staple Mesoamerican food and principal crops in ethnoagroforestry systems practiced by 21 cultural groups throughout Mexico (19 indigenous people) We recorded on average 121 ± 108 (SD) wild and domesticated plant species, 55 ± 27% (SD) of them being native species; 44 ± 23% of the plant species recorded provide food, some of them having also medicinal, firewood and fodder uses. A total of 684 animal species has been recorded (17 domestic and 667 wild species), mainly used as food (34%).

**Conclusions:**

Ethnoagroforestry an emergent research approach aspiring to establish bases for integrate forestry and agricultural diversity, soil, water, and cultural richness. Its main premise is that ethnoagroforestry may provide the bases for food sovereignty and sustainable ecosystem management.

## Background

The vast majority of the world’s biodiversity is located in the tropics [1]; but it is known that it is dramatically decreasing as long as people of the region significantly depend on it for their subsistence [1]. Conversion of forest to agricultural areas and pasturelands for cattle grazing are among the main causes of loss of biodiversity in the World [2], although more recently mining is progressively increasing its destructive impact in great areas. The traditional, indigenous, small-scale agriculture or peasant agriculture has been pointed as one main cause of poverty and hunger in the tropics, based on misunderstanding of peasant life patters, and ideological characterizations of these systems as low productivity systems, economically inefficient, unable to self-sufficiency and responsible of environmental degradation [2, 3]. However, the majority of the farmers in the global south are small-scale producers, practicing agriculture in a high variety of forms; therefore, the traditional agriculture and the relationship with biodiversity, poverty and hunger is also highly variable [1] and it cannot be a linear cause-effect conclusion. Poverty, hunger, marginalization of peasants, environmental degradation and biodiversity loss in these regions have a history more clearly linked to colonial and neoliberal policies, mining destruction of natural resources and ecosystems, industrial models of production in agriculture, livestock, forestry, oil and mineral extraction, and predatory policies of great corporation rather than responsibility of traditional agriculture [4].

Food sovereignty has emerged as a concept counter framing the corporative food regimes, broadly defined “as the right of the nations and peoples to control their food systems, including their own food cultures, production models according with their environments, their own forms of interchange and commerce” [5]. This concept has “re-appropriate [d] the term peasant and infuse [d] it with a new positively valued content” [5]. Local, traditional, indigenous, small-scale, or peasant agriculture or agroforestry have been considered as capable to sustain ever-growing demand of agricultural products while conserving biodiversity, providing critical ecosystem services, maintaining livelihoods and food sovereignty [6, 7].

Nearly 53.4% of the Mexican people live in conditions of poverty [8], and nearly 44% belong into categories of food insecurity [9], the higher percentage of them being rural and indigenous people [10]. Paradoxically, Mexico is a megadiverse country, with high biological and human cultural diversity, as well as high agro-biodiversity and diversity of agricultural systems constructed throughout the longest history of domestication and agriculture of the New World [11]. From such ancient and diverse interactions between people and local ecosystems and biotic resources has emerged one of the highest expressions of biocultural diversity of the planet [12, 13]. From such a context, *Ethnoagroforestry* has raised as a research approach looking for documenting, systematizing and understanding the brad and complex spectrum of forms of agricultural, ecosystems and landscapes management, integrated in local strategies for procuring food security and sovereignty. These strategies include the using of the wild, the whole ecosystems inside and around the agricultural plots, the species diversity of the whole system. In addition, it studies the diversity of management forms, including incipient and advanced form of management of elements of the systems [14]. In addition, the strategies include a great diversity of forms of management of biodiversity, including plants, animals, fungi and microbiota, wild, semi-domesticated or in advanced levels of domestication under diverse mechanisms of artificial selection [15, 16]. The *Ethnoagroforestry* approach aspires analyzing agroforestry management as part of particular human cultural contexts in which the productive systems are part of social life and economic relations. Either individuals or households, communities and cultural groups who have a leading role in directing the interactions and design or modeling the components of landscapes are all crucial for understanding the drivers of AFS [17–19].

The importance of local traditional agroforestry management of Mexico has been widely recognized [17]. However, a systematic analysis of the management experiences of Mexican agroforestry is still necessary in order to identify in a deeper detail the particular contexts where the systems can be successful and requirements for adapting and improving their use and management. Such understanding would contribute to stop the unfortunate losing of the *Cinderella* agroforestry systems, that is happening throughout the world. The *Cinderella* term makes reference to agroforestry systems unrecognized and forgotten at global level but with high relevance at regional and local scale for food production, environmental protection, conservation and recovering, and social wellbeing [20]. The process of enhancing agroforestry systems [21]. The main purpose of our review is analyzing: (1) how much biodiversity (plant and animal species richness and diversity) is maintained under ethnoagroforestry management, (2) what is the importance of such diversity for food sovereignty systems; and (3) what are the potential, challenges and limitations for integrating the ethnoagroforestry approach for achieving food sovereignty in México. We hypothesized that the ethnoagroforestry management forms may be the basis for food sufficiency and sovereignty in Mexican communities, regions and the whole nation and that the routes of technological innovation according to the contemporary social needs are identifiable and possible to be attended based on local and regional TEK and agroecology and agroforestry criteria.

## Methods

### Construction and use of databases

We conducted an exhaustive search of information about, agroforestry systems, management strategies, their biodiversity conservation capacity, their components and roles in social life of people managing the systems, their economic capacity among other topics summarized in Table 1. Systematization and analysis of information reported in this study was conducted from August 2011 to May 2015, but the database is still in construction. We constructed the database *Ethnoagroforestry* based on i) our own researches, ii) by consulting Google Scholar, Scopus, Redialyc and SIDALC databases; iii) Mexican and international specialized journals in agroforestry, ethnoecology and traditional ecological knowledge; iv) catalogues, libraries and online available information from universities and research centers; and unpublished theses.

**Table 1:** Topics analyzed in database for this research

Topic	Description	Types
System type	It is the classification of types of ethnoagroforestry systems in relation to Moreno-Calles et al. 2013 y 2014.	Homegarden, agroforest, long fallow agroforestry, arid and semiarid agroforestry, terrace agroforestry, wetland agroforestry system, agrosilvopastoral system.
Reference	It is the reference to the work, year and author.	Papers, book, thesis, databases.
Place	It is the place where the work was done. The information is reported only when it was available in the paper	State, municipality, town.
Cultural group	It refers to the name of the group of people originating or name that cultural group that manages the system is reported	Mayas, nahuas, mixtecos, mixes, totonacos, triquis, mazatecos, otomies, tzeltales, teenek, chontales, popolucas, zoques, raramurís, tojolabales, tzotziles, tepehuanos, zapotecos, tlahuicas, ixcatecos, rancheros.
Climate and vegetation type	Include the type of climate and associated vegetation when the information was available.	Climate: Arid, semiarid, subhumid, template, tropical.
Local or regional system name	It is the local or regionally name of ethnoagroforestry system in the original or in Spanish language.	Examples: *kuojtakiloyan*, *te’lom*, *calmil*, *ekuaro*, *kool*, *tlacolol*, *metepantle*, *coaxustle*, *calal*, *chinampa*, *milpa-chichipera*, *huamil*.
Species number	Is the number of species reported in the studies reviewed or calculation from biodiversity inventory.	Local level (one community), regional level (two or more communities)
Native species	Is the number of species native to Mexico and the percentage of them according to the percentage of species reported with respect to the identified native species.	Native to México Introduced from other country
Uses and benefits types	The uses are reported in documents consulted and standardized according to a classification of uses for agroforestry systems in plants built	Plants uses (17 uses): food, medicinal, ornamental, firewood, fodder, construction, crafts, fibers, toys, envelope, cosmetic, aromatic, tools, resin and latex, colorant, poison, hygiene.
	by Moreno-Calles et al. 2012 and 2013. For animal classification uses and benefits were built in this work. The benefits identified for agriculture, forestry, home economics or the environment are also reported.	Plants benefits (19 benefits): habitat or food for useful species, pest control, improving the climate, maintaining of water sources, storage crops, improving soil fertility, soil retention, shadow, windbreak, hurricane protection, fire control, attractor rain, land delimitation, vegetation recovery, environmental indicator, rituals, barter or sale, hedgerow. Animals uses (8): food, fertilizer, aquaculture, hunting, medicinal, protection, recreative, gift. Animal benefits (7): melliferous, rituals, work, transport, polination, pest control, barter or sale.
Main crops	It refers to the main crop or crops are reported in the work reviewed.	Native maize, beans, pumpkins, coffee, cocoa, pineapple.

**Fig. 1:**
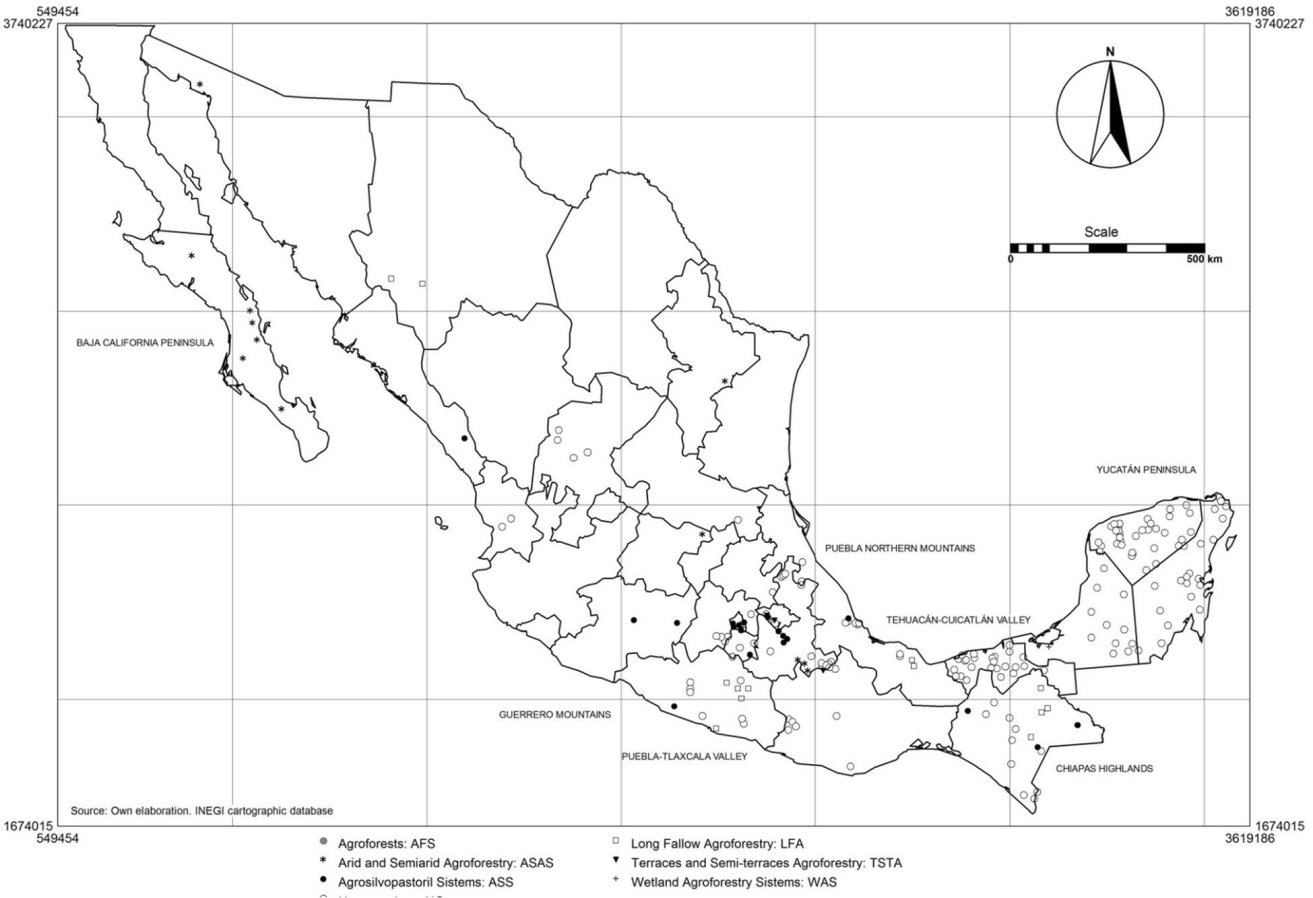
Ethnoagroforestry systems in México. Municipalities where studies about plant and animal biodiversity had been realized. Principal regions mentioned in the paper

The keywords included in the search were: agroforestry and Mexico, agroforestry system and Mexico, traditional agroforestry system and Mexico, traditional agriculture and Mexico, trees in agricultural plots and Mexico, agroecosystem and Mexico, agroforestry practices and Mexico, hedges plants and Mexico, living fences and Mexico, small farm agriculture and Mexico with quotation marks (Table 1). Searches that are more specialized were also conducted on the regional or local names of different agroforestry systems and agroforestry practices documented in a recent review of the systems by Moreno-Calles et al. [17] in English, Spanish and original language: (1) Homegarden, “huerto familiar”, *calmil*, *ekuaro*,“solar”; (2) Agroforest, “agrobosques”, *kuojtakiloyan*, *te’lom, cacaotal*, “café bajo sombra”, “piñal”; (3) Long Fallow Agroforestry: “roza, tumba y quema”, *tlacolol*, *kool*, “agricultura itinerante”, “slash and burn agriculture”, “shifting agriculture”, “swidden agriculture”; (4) Arid and Semiarid Agroforestry, “sistemas agroforestales de zonas áridas”, *milpa-chichipera*, *garambullal*, *jiotillal*, *huamil*, *coaxustles*, “oasis”, “desert garden”*, tajos*; (5) Agroforestry Terraces, “terrazas”, “semiterrazas”, *metepantle*, “terrace agriculture”, “terracing”, “sloping field”; and (6) Wetland Agroforestry Systems, “sistemas de humedales”, “agricultura de campos elevados”, *calal*, *chinampa*, “campos drenados”, “drained field”, “raised field”. We included records on agrosilvopastoral systems specifically for this review because economic and environmental relevance for traditional agroforestry in Mexico. In total, we collected 740 references about agroforestry systems and organized the information in the database *Sistemas Agroforestales Tradicionales de México*. But only 192 papers, books and theses have been collected in relation to this research. The following criteria for the inclusion of the reports in the analysis richness were also considered: i) reports by an author for the same locality, taking into account the latest report; dissertations are reported in cases in which there is no any publication about a study; ii) only papers including inventories of wealth in the main text. The documents that included inventories about plant species richness were in total 148 (Appendix 1) and 44 reporting inventories of animal species (Appendix 2). Information from these references was systematized and analyzed, including maps indicating locations of the different AFS of Mexico, which were determined from the review of documents, and crossing the information with the database of municipalities from INEGI [22]. The data processing was performed with the geographic information system Ilwis open (Fig. 1). In addition, we conducted a review of databases using the key words “food”, “food security”, “food sovereignty” and “local food system” and agroforestry and Mexico. Food sovereignty papers are particularly examined in the Discussion section.

### Diversity analysis

Agroforestry systems and their contribution to understand the conformation of landscapes were analyzed by grouping AFS into seven categories based on the typology built by Moreno- Calles et al. [17]. The number of reports about these systems, the scale of the studies (regional or local), their geographical localization, the cultural group that manage them, the general characterization of environmental conditions, the agroforestry practices, local names, main crops cultivated, agricultural techniques, forestry management, among other issues were registered (Table 1). The studies have been separated into local (one community) and regional scale (several communities) to the calculation of average species richness and standard deviation.

The information about biodiversity included family and species categories, and records were included into flora and fauna databases, ecological information such as average richness of plants and animals was calculated. A classification of plants and animals’ species uses and benefits was constructed by calculating species percentage used as food and other uses, mainly medicine, fuelwood, and others related with the food system.

## Results and discussion

### Forestry and agricultural diversity and their multiple uses and benefits

#### Homegardens (HG)

Integrated in this category is a great variety of agroforestry systems characterized by their multi-strata plant composition, managed intensively attached to or near the households’ homes. In these systems, a high number of wild and domesticated perennial and annual plant species with different uses and often domestic animals are let standing, transplanted, cultivated and cared. Studies at global level carried out in several countries show that households practice this system for food production for subsistence or small-scale marketing and the variety of crops and wild plants provides nutritional benefits [23]. Homegarden is by far the type of agroforestry system with the largest number of studies, in Mexico as well as in the whole world [24]. In México, 95 HG studies provide good inventories of plant species maintained in there. These forms of management are reported for 20 states of the country, in temperate, tropical, sub-humid, arid and semi-arid climate conditions. The plant richness is on average 122 (±95) species in local home garden studies, and 279 (±143) species in diagnoses at regional level. On average, 56% of the species recorded in the studies of homegardens are native plant species. According to the studies reporting this information, HG have mostly species uses as food (46% ±24), in similar proportion with ornamental plant species. Both groups of species are destined to direct consumption (the edible ones) and spiritual satisfaction (the ornamental ones), but a small portion of products are commercialized, bartered or given as gifts to friends. Nearly 24% of the plant species recorded is used as medicine. Then, the following most common species are those used as fodder (10%), honey producing plants, and fuel (8%), soil retention, live fences, habitat or facilitator of valuable species and pest control (≤5%). Similar results were reported by Caballero et al. [25], who documented nearly 1400 plant species occurring in Mexican HG, 572 of them (nearly 41%) being medicinal species, 528 (37.7%) ornamental, 442 edible (31.6%) and 682 (48.7%) plant species having other uses. Studies of HG from the Yucatán Peninsula compiled by Guido [26–28] provide relevant information about animals and plants of that region. These authors report 572 plant species with ornamental, food, medicinal and honey production, as the main uses. Among the most important studies with animal biodiversity are those by Mariaca et al. [29], who noted the wealth of wild and domesticated animals in the 200 homegardens sampled in southeastern México with an inventory of 30 species of wild and 17 domestic animals only for that region. Our review identified 13 studies recording the presence of 148 animal species, 131 of them being wild and 17 domestic. The dominant groups were birds (89 species), followed by mammals (43 species), reptiles (12 species) and insects (4 species). We recorded nearly 20 benefits provided by animals maintained in homegardens, mainly food (20%), ornamental (17%), recreational (17%), pollination (9%), raising for trading and other uses such as labor and transportation (7%), medicinal uses, weed and pest controllers, providers of fertilizer, ritual, and protection (>5%).

#### Agroforests (AGF)

These are spaces where peoples manage vegetation in order to change its composition according to their purposes and needs, preserving attributes and functions similar to those of the natural forest [30]. Some of these systems have been recorded with the name of *acahuales*, a term that more commonly refer to fallow areas. In several regions of Mexico, fallow areas are managed by enriching their composition with wild, weedy and even domesticated plants. AGF may be located close to the house, as a kind of variation of homegardens or may be fallow areas of slash and burn systems after cultivating maize, beans and squash. In addition, agroforests may be integrated into single management unit areas with managed *acahuales* and large fallow patches. Agroforests in México are complex integrated forms of landscape management and agroforestry systems where the main crops include growing species such as coffee, cocoa, pepper, vanilla, pineapple and crops of local relevance for consumption such as maize, beans, sugar cane, and citric species, among others. This form of management is recorded in the literature in eight states of Mexico, including Puebla, Oaxaca, Chiapas, Tabasco, Guerrero, Veracruz, Jalisco and Nayarit, all of them with warm and sub-humid tropical conditions and possibility the *pet kot* can be a type of Mayan agroforest in Yucatán and Quintana Roo linked to wild and domestic animal management [31]. Agroforests are the second more registered agroforestry system in the literature in Mexico (112 papers). Among the most relevant reviews about biodiversity in agroforests are those for the *te’lom* [32] and the *kuojtakiloyan* by Martínez-Alfaro et al. [33]. We reviewed 21 studies reporting plant species richness and 17 for animal species. In total, plant species richness of these systems is 1072 species and 414 animal species. This form of land management has a high percentage (67%) of plant species native to Mexico. However, the average data describe that not all agroforests have the same contribution to the total wealth. On average, each system unit has 55 (±31) plant species and 266 (±75) species at regional level. The main uses and benefits of plant species includes 20 different types, among them the most important are food (53%), medicine (18%), firewood (12%), timber and construction (8%), ornamental (4%), and other uses. For animals, 17 of the studies emphasize that wild birds are the main group (228 species), followed by mammals (90 species), insects (38 species), reptiles (30 species) and amphibians (28 species). Studies in coffee agroforests report of edible insects three species [34], but this group has been poorly studied.

#### Long fallow agroforestry (LFA)

These extent systems are recognized by the long fallow period in relation to the period of land cultivation and by alternating use and fallow periods [34, 35]. These systems are more commonly known by the method of thinning and clearing natural vegetation in order to make space to crops and have been named slash and burn or swidden agricultural systems. These extensive systems practice mainly rainfed agriculture, where maize, beans and squash are grown. The landscapes which are part of these systems include patches of forest, agroforests or *acahuales* or fallow areas used for producing coffee, pepper or sugar cane. Also common is the presence of agrosilvopastoral and homegardens systems in the agroforestry landscapes. Currently, systems of long fallow are distributed in the mountainous terrain of steep slopes of México, mainly the tropical deciduous and temperate forests of the states of Chiapas, Chihuahua, Guerrero, Jalisco, Michoacán, Morelos, Nayarit, Oaxaca, and Puebla. However, this system is also common in the flat or gentle slopes areas of the tropical forests with thin and poor calcareous soils of the Yucatán Peninsula. Local names of this system may include: *tlacolol* in the mountains of Guerrero [36, 37], the Maya milpa (*kool*) of the Yucatán Peninsula [38, 39], the *mawechi* of the Sierra Tarahumara [40], the *coamil* in Jalisco [41] and Colima [42], the *huamil* in the coast of Michoacán [43] and *pot’kkan* in Oaxaca [44] Only eight studies provide information about plant species richness. According to the average data, these systems are able to maintain on average 142 ± 108 species (SD). Most plant species in these systems have medicinal uses (51%), but others are food (26%), firewood (18%), construction (12%) and living fences (6%). In three studies the authors reported that fauna in these AFS includes 46 species, mainly mammals (28 species), birds (12 species), insects (4 species) and reptiles (2 species), but exhaustive inventories are clearly needed. Information about the relevance of these systems for hunting and importance for food is illustrated by recent papers [45], which report that Mayan people cultivate milpa with the purpose of attracting animals for hunting. Similarity, Bernice [46] had previously documented that early secondary forests are attractive spaces for animal species valued by the Maya like the ocellated turkey, deer and peccary.

#### Arid and semiarid agroforestry (ASAS)

Arid and semiarid areas are characterized by a high risk and uncertainty of agriculture and other productive activities [7]. Management of soil, water, and vegetation cover has been important in the development of sustainable agroforestry systems. These areas are described as semi-intensive agroforestry systems mainly settled on slopes of rocky areas dominated by prickly pears forests, the *huamil* in the Valley of Santiago, Guanajuato [47, 48]. Also, in landscapes with terraces dominated by species of maguey (mainly *Agave salmiana*), created on the slopes and foot slopes of Valley of Mezquital, Hidalgo [7], in cacti and *izotal* forests in the Tehuacán Valley [49–51]. These forms of management may also have carried out in conditions of seasonal access to water, as it is the case of natural or created areas adjacent to rivers [52] and in the ravines. In alluvial areas, people have of created complex systems terraces locally called *coaxustles* in the Tehuacan Valley, and *tajos* on the banks of the rivers of the Sierra Gorda, at Xichú, Guanajuato [53]. Some of these systems can also occur under conditions of permanent access to water like the development and establishment of agroforestry oases in Baja California [54] and the *desert gardens* in San Luis Potosi [55]. Also relevant are the homegardens under semiarid conditions of the Tehuacan Valley [56]. These are the systems with the least number of reports recorded in the literature in arid and semi-arid AFS in seven states of México (23 papers), only nine of them providing information about species richness and uses. These studies have emphasized different life forms (trees/shrubs/herbs), but sample sizes and methods are different and difficult to compare. However, it is possible to identify that in arid and semiarid agroforestry systems people maintain on average 69 ± 33 species of plants (SD), 71% of them native species and in regional reports are 90 (±38) species. In almost all these systems, maize is the main crop in combination with beans, squashes, chili peppers and other edible species like peanuts, watermelon, melon, tomatoes and amaranth. However, it can be identified that as there is greater availability and access to water, people prefer to cultivate introduced species for commercialization, decreasing the percentage of native species present in agroforestry systems and those used for direct consumption. Plant species present in the form of managed are used as food, mainly for the production of edible fruit and flowers (35%); fruits are consumed fresh, in jams, liqueurs, nuts and even are exchanged or sold for obtaining other resources, whether in the community or in regional markets [57]. Other uses include fodder (25%), shade (17%), firewood (16%), and as retainers of soil and water as well as borders and living fences (15%), ornamental (12%) and wood (10%). Minor uses include ceremonial, handcrafts, habitat for edible animals, stock, alcoholic drinks (≥5%). Wildlife studies are still scarce, and those available recorded 97 species, mainly birds (78 species), wild mammals (14 species) and insects (5 species). The principal uses include food (9 species), pollination (6 species) and ritual (3 species). There are very important edible species of insects that have been documented in ethnoentomological studies in semi-arid areas. Edible insects are generally reported to be in interaction with trees, shrubs, prickly pears, cacti and agaves which are tolerated, encouraged, protected and cultivated in agroforestry systems mainly in order to favor the availability of edible insects, particularly larvae of Lepidoptera, Hymenoptera and adult Hemiptera [58]. Host plants of edible insects are deliberately managed in AFS in order to get these resources for direct consumption in households or for trading them.

#### Terraces and semi-terraces agroforestry (TSTA)

Actions to maintain soil fertility, moisture and to decrease the effect of frost on agricultural systems are common concerns for farmers and one way to achieve it is the construction of terraces [59, 60]. However, it is important to notice that not all terraces are agroforestry systems, because only some of them include the management of wild and domestic components on the terrace borders or walls, either as a way of strengthening the terrace or because of other uses of the species. In these forms of management that are located mainly under temperate and semi-arid conditions, maize, beans and squash as main crops are grown, although recently in temperate zones people are growing alfalfa, potatoes, barley and other crops. We reviewed 25 papers documenting this system, but only four of them provide information on plant species richness and use. The average number of species recorded is 51 ± 42 species. (SD) The principal uses include medicine (40%), food (19%), firewood (20%), soil and water control (25%), handcraft, ritual and fodder (≥5%). Among the species most commonly used in terraces of temperate zones are prickly pears, used for consumption of cladodes, fruits and edible seeds and several species of the genus *Agave*, which are valued for producing the sweet sap *aguamiel* and the fermented sap called *pulque* [59, 61–64].

#### Wetland agroforestry systems

These are systems in which the soil is raised above the water level, using materials such as mud, organic matter, trees, clusters of vegetation among others materials, in order to stabilize a portion of land as a kind of isle. Water is drained by channels and such systems are known in the literature as raised-field or drained agricultural fields [65]. Few of them remain active in México, the best known are the called *chinampas* of the Valley of México, the ridges or *calales* in the southwest of Tlaxcala [66] and the *camellones chontales* in Tabasco [67]. These systems have been of great academic interest since they are considered the most intensive systems of ancient México that currently persist. These agroforestry systems are extraordinarily fertile and productive due to the soils rich in organic matter, which allow them to nourish a high density and variety of crops that have been able to sustain large human populations [68]. The number of reports recording this management form is 51, but only six of them provide information on the wild and domestic diversity, most of the works have emphasized agricultural diversity. The average plant species recorded is 56 ± 44.20 species (SD), the main uses of these species are food (54%), ornamental (43%), handicraft (24%), fertilizer (24%), living fence (11%), and windbreak (8%). The main crops for human consumption are vegetables, aromatic herbs, fruit trees and to a lesser extent legumes and grains [68]. Several species of aquatic plants are exploited for human consumption [66]. Only four papers provide information about animals, which record 89 animal species, mainly fishes (32 species), birds (25 species), reptiles (13 species), malacostracans (8 species), mammals (5 species), amphibians (3 species), chondrichthyans (1 species), gastropods and other mollusks (2 species). The 63% of the species recorded are used as food.

#### Agrosilvopastoral systems (ASPS)

Although presence of domestic animals is a general feature of peasant systems, there are ways of handling systems that are explicitly pastoral where the animal component is central in the management purposes, and these are integrated parts of agricultural and silvicultural management. This agroforestry system type is common in temperate, tropical and semiarid zones. We reviewed 15 studies conducted in Mestizo, Tzotzil and Zoque localities of Chiapas, Colima, Puebla, Sinaloa, Michoacán, México City, and Veracruz. All of them make explicit reference to domestic animals managed and the potential management of wild animals such as deer as alternative productive system. Although only five studies provide information on the species, and only for three case studies, species distributed in ASPS were specified. Animals commonly registered in this form of management include cattle, sheep, goats, donkeys, horses and mules, raised as source of food, power and fertilizer for agricultural activities and funding for emergencies. The vast majority of systems besides the animals’ handle crops such as maize, coffee, beans, squash, citrus, oats, sorghum, and grasses. Although no systematic inventories of plants were reported in any of these papers, we identified 44 plant species recorded in these systems. Most of the species mentioned are live fences, fodder, food, firewood, medicinal uses, and shadow for cattle.

### Potential ethnoagroforestry contribution to food sovereignty

#### Biocultural and ecological contribution

**Fig. 2:**
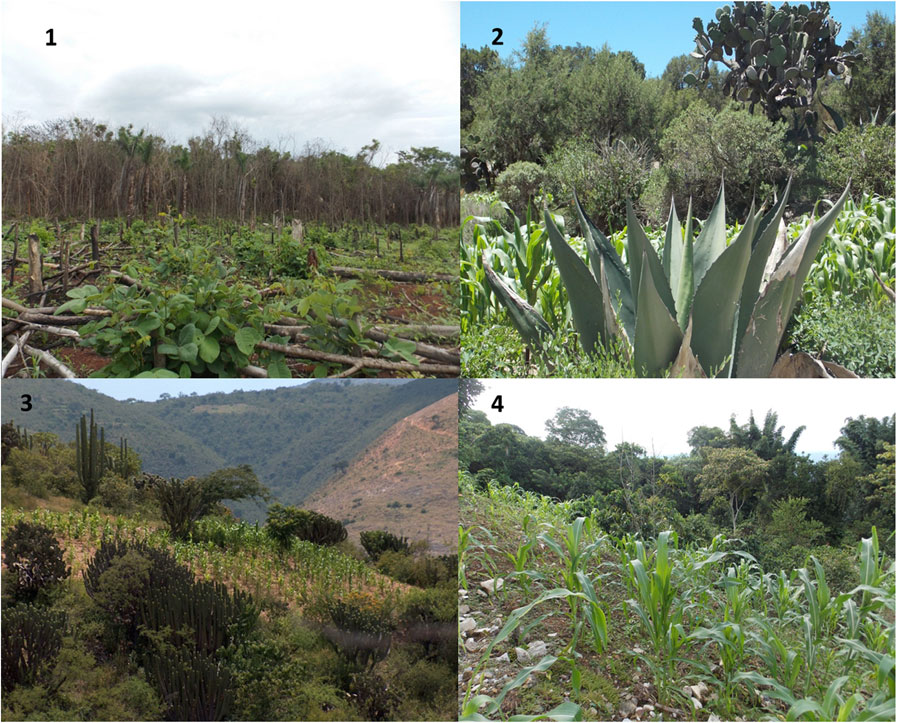
Agroforestry System in México. 1) Long fallow agroforestry; 2) Terrace agroforestry; 3) Semiarid agroforestry; 4) Agroforest and milpa

**Fig. 3:**
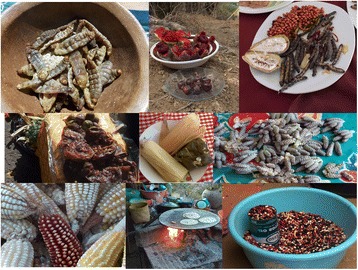
Food and other resources from Ethnoagroforestry Systems of México

According to our review, traditional agroforestry systems (Fig. 2) differ in their contribution to the maintenance of plant diversity and wildlife and domestic animals. It is still difficult to establish generalizations, since the contexts, purposes, plot and sample sizes evaluated were different among the systematized studies, however it is possible to identify some general patterns. It is possible to estimate that the 148 records of ethnoagroforestry systems reviewed maintain on average 121 ± 108 (SD) wild and domesticated plant species and 55 ± 27% (SD) of them are on average native. These species have as main use its consumption as food (44%) mainly fruits, flowers and leaves used as vegetables and spices, or for preparing nutritional drinks or infusions (Fig. 3). These species also have other important uses such as firewood and soil and water conservation, all crucial for food processing and without which it would not be produced the vast majority of what is consumed and which constitutes part of the triad suggested by Altieri et al. [69] as part of food sovereignty (energy, technology). In the case of animals, reports describe 684 species (17 domestic and 667 wild), with the principal use is being food (34%) and other 17 more uses like ornamental, recreation, transportation and for agricultural labor.

Traditional agroecosystems have not only provided to people resources for subsistence, such as food, medicine, and cash incomes, but have played an important role in biodiversity conservation, especially for conserving local species and native crop varieties and germplasm [70]. In México, local agroforestry systems contribute to local food systems and food sovereignty as long as they provide products directed to satisfy the demand of ingredients for local food. These systems are also settings where a great diversity of native varieties of maize, beans and pumpkins, the staple crops are managed, selected and diversified. One first step in the understanding of food sovereignty systems is knowing the local food systems; the inventory of the diversity of actual and potential edible resources available, their nutritional contribution, their cultural meanings and the surrounding context, including the ecosystems, landscapes, agroforestry systems, species and varieties used in local food systems [71].

Landscapes in México are composed by of multiple forestry, agriculture and agroforestry systems types, and this guarantee high biodiversity but also diversity in cultural landform management [29]. The main contribution of ethnoagroforestry handling for food sovereignty is the integration of biological and cultural, wild and domestic diversity, at different scales and forms of land management that allow synergy between objectives apparently recognized as opposites: the use and conservation of the biodiversity. The challenge of food sovereignty requires the integration of all bodies of wisdoms, knowledge and practices around the biocultural diversity (human diversity, landscape diversity, agricultural diversity, forest diversity, livestock diversity, wildlife diversity, soil diversity, water diversity, gastronomic diversity, energy diversity, climate diversity). A significant pattern is that as the sample size increases in the studies analyzed the greater wealth of species of plants and animals are incorporated, allowing ensuring conservation of forest and agricultural diversity at landscape scale, as well as at communitarian territory and region, due to the heterogeneity of the households’ contributions to the configuration of plots managed through ethnoagroforestry. Homegardens and long fallow lands are mainly used to secure food for home consumption, whereas coffee forest gardens (agroforest) are mainly used to generate cash income [72]. Complex landscapes and management systems may produce major species diversity and more complex food systems and vice versa through spatial and temporal diversity and heterogeneity in diet and landscapes [73]. Diversity of human culture enrich local and global productive and food systems with meanings, beliefs, wisdoms, knowledge and management practices “that is good for eat is god for think before” [74]. In addition, these elements establish the bases for constructing sustainable agroecological management systems [69] for local and global alternatives for food systems [71].

In México, 80% of forests (55.3 million hectares) are owned by 30,000 traditional communities and ejidos [75], and 81% of rural economic units are agricultural households (SAGARPA-FAO 2012). Additionally, 14.6 millions of people are recognized by themselves to be indigenous [76], distributed mainly in the center, south and southeastof the country . Agroforestry systems are practiced mainly in “communal” and “ejido” land in the main indigenous areas of México, where decisions are made through local assemblies, which are important institutions for constructing sovereignty processes in relation to access to land, territories, technology and resources. Food sovereignty is the right of nations and people for controlling their own food systems means of production, environments, food cultures and markets [77]. In all these processes, the communal and ejidal assemblies play crucial roles. The connections between food and nutrition security, among indigenous people and the preservation of cultural and biological diversity have been recognized in the “Declaration on the Rights of the Indigenous Peoples” [71]. Many environmental movements of México occur in the distribution area of ethnoagroforestry management zones [78], which indicates that indigenous people are the main promoters of food sovereignty. The organization *Tosepan Titataniske*, Vicente Guerrero A.C organization, and Grupo de Estudios Ambientales and the *Sansekan Tinemi* organization and communities are Mexican examples of movements in defense of land, seeds, water, environment and autonomy all elements linked to food sovereignty [79].

In addition to the socio-political contributions [80], the relevance of the concept of food sovereignty compared to the one of food security, promotes ethical reflection on how interactions among people and how people can live together with other humans and other living things to meet the human needs, in the case analyzed food. By placing the emphasis on the ethical implications of current forms of production and consumption there have been drawn questions about the best ways to act without affecting the right the decision processes on diversity, production, access and distribution of food. Similarly, the discussion and decision about the involvement of world views, livelihoods and cultural diversity that until recently were relevant in many humans for food stocks and that are now recognized as “outdated views and wasted lives” [81] and production systems that are part of the agroforestry systems reviewed. The relationship between wasting food and hunger in the world should be re-thinking [82], as well as the effects of production and food systems in nonhuman living, good water, air and soil and the environment in general and their implications for the quality of life and human health [83–85].

### Limitations and challenges of traditional agroforestry for food security and sovereignty

However, the need of integrating biodiversity management, food production systems, food local systems and food security and sovereignty, which are complex issues affecting globally human beings, until recently these issues were addressed separately, emphasizing the importance of research of either social or natural processes instead of integrating both science and social actions for solving food insecurity problems. In the analysis of information of this paper, it is notorious the scarcity of studies on biodiversity productive systems with sovereignty and food security or local food systems concepts (<10% of studies analyzed). Nevertheless, nearly 80% of the reports reviewed mention the relevance of agroforestry systems for self-sufficiency of food, medicinal or firewood for cooking. It is relevant that among the wild and weedy components of agroforestry systems, edible plants include numerous species of *quelites*, the traditional greens that are known to provide important vitamins and fiber to diet, fruits and nuts providing vitamins, proteins and oils, as well as some roots and tubers that contribute with starch and fiber [86]. It is also relevant to mention that numerous species of insects are deliberately protected for ensuring their consumption, which together with hunting animals, are relevant sources of proteins for the traditional diet.

How can we explain the presence of hunger in places where these forms of management are practiced in association with a high biological and cultural diversity? What kind of socioeconomic, politic and cultural processes do not allow access to biological and food diversity? The principal obstacle to use local biodiversity for local food systems is poverty together with discrimination to indigenous food. In many cases studied, people prefer to sale a good food from local biodiversity and with the money paying other needs like, health care and child education. Maize production or other relevant crops for food are commonly insufficient, especially in arid and semiarid zones, where people only produced one third of the annual maize needed for food and fodder and under drought only fodder and seeds for next year [50]. Either staple crops like native maize or wildlife resources of good quality are sold to have economic profit, even if they have to buy other products of lower quality as industrial corn [87]. Another important limitation relates to the abandonment of the consumption of traditional food of high quality and nutritional and cultural value in the past, but that currently are used as fodder or uses other than human consumption, for example the case of *ramon* (*Brosimum alicastrum*) in Mayan homegardens [88].

Nowadays, the world faces important dilemmas about the need to preserve biodiversity and ecosystems benefits, at the same time that producing enough food for actual and future generations. The tremendous impact that the modern technology for producing food has caused on natural ecosystems in only few decades indicates that continuing that route is inviable; in other words, it is important recognizing the unviability of maintaining or increasing the rhythms of agricultural production under the technological models predominating throughout the world. Small farms systems cover an important surface of the areas of the world dedicated to produce food, but most of them do not work in the logic of high productivity as agro-industries do. Such small farms are reservoirs of biodiversity, valuable genetic resources and traditional ecological knowledge constructed throughout thousands of years of agricultural experience. All these elements have important signs of local adaptations to attend the local needs through local techniques. But the preliminary systematizations of knowledge and techniques involved in these systems indicate the occurrence of similar principles in common: the importance of maintaining diversity, soils and availability of water. Numerous technological expressions of these principles reveal that the traditional ecological knowledge has functioned with similar motives in different ecological and cultural contexts. Such traditional ecological knowledge and praxis is currently a valuable source of technological options to develop innovations needed to adapt the small farms to the current needs of producing food and other raw matters following sustainable principles. It is a human experience constructed for millennia that deserves to be understood and systematized. Agroecology and Ethnoagroforestry has a high responsibility to construct alternatives to the failed agro-industrial production models, and has in TEK an important source of knowledge and techniques to construct the innovations required for a world that cannot be supported by the disasters caused by the agro-industries.

## Conclusions

Ethnoagroforestry complex are still alive; they maintain staple crops and a high diversity of plant and animal resources, in addition to fungi and microbiota scarcely analyzed. These systems are important reservoirs of biological diversity that is directly consumed as food and complement other needs of the food system as medicines, fuel and other goods and benefits. These systems have enormous advantages in terms of conserving biodiversity, ecosystems integrity and providing resources to people. The main challenges however are their preservation. Several factors associated to the modernity and intensive agricultural systems counteract against these systems. Land tenure is progressively fragmenting and intensification of land use is also increasingly displacing the previous traditional systems maintaining forest cover. However, the systems and people that have driven them are real and their experience is still to be documented. It is not a question of agricultural technique and biodiversity conservation but also a question of human culture and the rationality of a way of living and deciding what to eat and how to eat. These are the elementary bases of food security and sovereignty and basic source of knowledge and techniques for constructing agroecological and ethnoagroforestry innovations for sustainable forms of producing food and raw matters.

## Appendix 1

**Table 2:** Reports by agroforestry system type in Mexico with plant species inventory

Author	State	Municipality	Environment	Native people	Number sampling units	Number of species	Percentage edible species (%)
HOMEGARDENS							
Local Reports							
Acosta et al. (2012)	Yucatán	Valladolid	Sub-humid	Maya	54 plots, 29 milpas, 12 on the road, 7 on the forest	82	ND
Aguilar et al. (2009)	Oaxaca	Candelaria Loxicha	Tempered	Mestizo	31 homegardens	223	43
Aguilar-Cordero et al. (2012)	Yucatán	Mérida	Sub-humid	Mestizo	ND	28	59
Aguilar-Nah et al. (2012)	Campeche	Halachó	Sub-humid	Mestizo	3 homegardens	112	ND
Alayon-Gamboa (2010)	Campeche	Calakmul	Tropical	Mestizo	ND	31	3
Álvarez-Lugo (1997)	Veracruz	San Andrés Tuxtla	Tropical	Mestizo	4 homegardens	88	ND
Ángel-Pérez y Alfonso (2004)	Veracruz	Coxquihui	Sub-humid	Totonaco	40 homegardens	223	33
Ávila et al. (2012)	Yucatán	Mérida	Sub-humid	Mestizo	21 homegardens	187	ND
Barrera (1999)	Guerrero	Copalillo	Tempered	Mestizo/nahua	ND	7	100
Bautista-García et al. (2014)	Tabasco	Cárdenas	Tropical	Mestizo	29 homegardens	80	ND
Blanckaert et al. (2004)	Puebla	Coxcatlán	Arid	Mestizo	30 homegardens	233	30
Burgos-Lugo et al. (2012)	Yucatán	Maxcanú	Sub-humid	Maya	ND	120	21
Cano-Ramírez (2003)	Guerrero	Ayutla de los libre	Sub-humid	Mixteco	10 homegardens	129	39
Cano-Ramírez et al. (2012)	Estado de México	Ocuilan	Tempered	Tlahuica	33 homegardens	287	19
Carrasco-Hernández (2011)	Hidalgo	Cuautepec de Hinojosa	Tempered	Mestizo	8 homegardens	120	23
Castillo-Puc et al. (2012)	Yucatán	Izamal, Peto	Sub-humid	Maya	30 homegardens	54	ND
Cahuich-Campos (2012)	Campeche	Hopelchén	Sub-humid	Maya	34 homegardens	153	33
Chávez- Guzmán et al. (2012)	Península de Yucatán	Maxcanú	Sub-humid	Maya	ND	138	33
Chi (2012)	Campeche	Champotón	Sub-humid	Maya	12 homegardens	156	53
Contreras-Cordero et al. (2012)	Yucatán	Maní	Sub-humid	Maya	50 homegardens	38	100
De Clerk y Negreros (2000)	Quintana roo	ND	ND	Maya	80 homegardens	78	59
De la Cruz (2009)	Veracruz	Tihuatlán	Sub-humid	Mestizo	ND	149	53
Escobar-Hernández (2013)	Chiapas	Unión Juárez	Tempered	Mestizo	24 homegardens	109	35
Espejel (1993)	Puebla	San Juan Epatlán	Tempered	Mestizo	ND	60	43
Flores (2012)	Yucatán	Mérida	Sub-humid	Maya	25 homegardens	79	ND
Flores et al. (2012)	Yucatán	Abala	Sub-humid	Mestizo	26 homegardens	223	46
Flores, Balam y Schober (2012)	Yucatán	Abala	Sub-humid	Maya	ND	134	ND
Flores, Kantun-Balam, Ortiz y Lara (2012)	Yucatán	Mérida	Sub-humid	Mestizo	ND	8	ND
Gispert et al. (2012)	Morelos	Tlaltizapan	Tempered	Mestizo	10 homegardens	9	67
Gómez-Álvarez (2012)	Tabasco	Centro	Tropical	Mestizo	ND	112	ND
Gómez-García (2011)	Tabasco	Cárdenas	Tropical	Mestizo	ND	93	46
Gómez-Gómez (2010)	Tabasco	Cárdenas	Tropical	Mestizo	126 homegardens	127	ND
Gomez-Montes de Oca (2009)	Zacatecas	Fresnillo/Sombrerete	Arid	Mestizo	ND	140	11
González- Jiménez et al.	México	Malinalco/Tenancingo/Villa Guerrero	Tempered	Mestizo	60 homegardens	37	ND
Granados-Sánchez (1999)	Quintana roo	Carrillo Puerto	Sub-humid	Maya	ND	127	48
Guerrero-Leal (2014)	Tlaxcala	Españita	Tempered	Mestizo	45 homegardens	28	86
Gutiérrez-Barrera et al. (2012)	Yucatán	Mérida	Sub-humid	Mestizo	ND	17	ND
Gutiérrez-Miranda (2003)	Chiapas	San Fernando	Sub-humid	Mestizo	17 homegardens	208	33
Guzmán- Sánchez (2012)	Tabasco	Nacajuca	Sub-humid	Mestizo	17 homegardens	101	35
Hernández- Burela (2005)	Tabasco	Huimanguillo	Tropical	Mestizo	ND	148	32
Hernández-Ruiz (2013)	Oaxaca	San Pedro Ixtlahuaca	Tempered	ND	16 homegardens	67	31
Hernández-Soto (2009)	Puebla	Coxcatlán	Arid	Mestizo	ND	314	36
Herrera-Castro (1992)	Yucatán	Valladolid	Sub-humid	Maya	10 homegardens	387	12
Herrera-Castro (2012)	Veracruz	Zozocolco	Tempered	Totonaco	ND	24	ND
Herrera-Martínez (2010)	Oaxaca	Huautla de Jiménez	Tempered	Mazateco	7 homegardens	76	34
Lagunas Brito (1992)	Morelos	Zacualpan de Amilpas	Tempered	Mestizo	ND	62	48
Larios (2013)	Puebla	Coyomeapan	Tempered	Mestizo	30 homegardens	281	35
Lazos y Álvarez- Bullya (1988)	Veracruz	San Andrés Tuxtla	Tropical	Mestizo	64 homegardens	338	25
Mendoza-García (2011)	Veracruz	Paso de Ovejas/Veracruz	Tropical	Mestizo	ND	53	ND
Mezquita-Ruiz et al. (2012)	Yucatán	Tunkas	Sub-humid	Mestizo	33 homegardens	148	37
Moctezuma (2014)	Tlaxcala	Izamal	Tempered	ND	8 homegardens	28	49
Monroy y García (2013)	Morelos	San Francisco Tepeyanco	Tempered	Nahua	ND	23	65
Monroy y Vergara (2012)	Morelos	Puente de Ixtla	Tempered	Mestizo	30 homegardens	14	100
Morales-Cabrera (2006)	Guerrero	Puente de Ixtla	Sub-humid	Mestizo	ND	94	47
Neulinger et al. (2013)	Campeche	Calakmul	Sub-humid	Maya/mestizo	20 homegardens	310	35
Oble (2005)	Estado de México	Calakmul	Tempered	Mestizo	ND	65	46
Orizaba Tovar (2008)	Guerrero	Texcoco	Sub-humid	ND	26 homegardens	12	100
Ortiz-León (2012)	ND	San Miguel Totolapan	Tempered	ND	16 fincas		ND
Osorio-Mendoza (2007)	Guerrero	ND	Sub-humid	Mestizo	ND	99	45
Osornio et al. (1999)	Península de Yucatán	Ayutla de los Libres	Tropical	ND	ND	24	ND
Pérez- Ramírez (2012)	Tabasco	Tlacuilotepec	Tropical	ND	107 homegardens	145	ND
Perezgrovas (2011)	Chiapas	Venustiano Carranza	Tempered	Tzeltal	ND	15	33
Pérez-Ramírez et al. (2012)	Tabasco	ND	Tropical	ND	58 homegardens	180	ND
Poot-Pool et al. (2012)	Campeche	Hecelchakán	Tropical	Mestizo	24 homegardens	236	24
Puente Pardo et al. (2010)	Tabasco	Hecelchakán	Tropical	Mestizo	32 homegardens	56	ND
Ramos-Zapata et al. (2012)	Yucatán	Huimanguillo	Sub-humid	Mestizo	5 homegardens	8	ND
Rebollar Domínguez et al. (2008)	Quintana Roo	Mérida	Sub-humid	Maya	20 homegardens	43	59
Rico Gray et al. (1991)	Yucatán	Yaxcabá/Tixpéhual	Sub-humid	Mestizo	42 homegardens	301	30
Rivera Lozoya (2013)	San Luis Potosí	Mérida	Tempered	Teenek	18 homegardens	208	44
Roces et al. (1989)	Veracruz	Aquismón	Tropical	Mestizo	8 homegardens	338	ND
Ruenes Morales (1993)	Nayarit	San Andres Tuxtla	Tempered	Mestizo	10 homegardens	201	27
Salgado Mora (2010)	Chiapas	Huehuetan/Tuxtla Chico	Tempered	Mestizo	24 homegardens	123	67
Sánchez Velázquez (2008)	Puebla	Santo Domingo Huehuetlán El Grande	Arid	Mestizo	10 homegardens	199	26
Santoyo (2004)	Puebla	Tehuacán	Arid	Mestizo	ND	90	35
Solís Becerra (2013)	Chiapas	Teopisca	Tempered	Mestizo	3 homegardens	13	100
Tamayo Ortega (1985)	Tabasco	Comalcalco	Tropical	Chontal	9 homegardens	242	37
Torres Díaz (2011)	Chiapas	La trinitaria	Tempered	Mestizo	30 homegardens	133	58
Torres Rosas (2010)	Tabasco	Cárdenas	Tropical	Mestizo	6 homegardens	130	47
Vásquez García (2007)	Veracruz	Mecayapan, Soteapan	Tempered	Nahua/popoluca	ND	28	100
Vázquez Medina (2010)	Puebla	Coyomeapan	Tempered	Nahua	ND	51	ND
Vibrans et al. (2001)	Estado de México	Texcoco	Tempered	Mestizo	20 homegardens	303	ND
Vilamajó et al. (2011)	Chiapas	Rayón	Tempered	Zoque	10 homegardens	63	36
Zaragoza et al. (2011)	Chiapas	Chamula	Tempered		ND	31	81
Regional reports							
Cámara-Córdova (2012)	Tabasco	Todo el estado	Tropical	ND	ND	141	ND
Cetz-Zapata et al. (2012)	Yucatán	ND	Sub-humid	Mestizo/maya	97 homegardens	86	ND
Chablé-Pascal et al. (2015)	Tabasco	Huimanguillo/Cárdenas/Comalcalco	Tropical	Mestizo	27 homegardens	330	42
Flores, Dillword y Kantun-Balam (2012)	Península de Yucatán	Cancún, Isla Contoy, Isla Mujeres, Tulum, Playa del Carmen, Isla Holbox, Isla Blanca, Cayo Sucio, Banco Chinchorro	Tropical	Mestizo	ND	96	ND
Flores, Garrido, Ortiz y Santos (2012)	Península de Yucatán	ND	Sub-humid	Mestizo	15 homegardens	265	ND
Flores-Guido (2012)	Península de Yucatán	Área Maya de la Península de Yucatán	Sub-humid		300 homegardens	524	15
García de Miguel (1998)	Península de Yucatán	Abala, Temax, Ucu, Calkini (Nunkini), Campeche (Hampolol), Tenabo, Maní, Tzucacab, Jóse Maria Morelos (Dziuche), Lázaro Cárdenas (Kantunilkin), Sucila, Tizimin (Tixcancal), Felipe Carrillo Puerto (Tihosuco), Chemax, Chichimila	Sub-humid	Maya	300 homegardens	156	59
García-Ramos (2010)	Oaxaca	Constancia del Rosario/Putla Villa de Guerrero/San Andrés Cabecera Nueva/Santa María Zacatepec	Tempered	Triqui/mixteco/mestizo	13 homegardens	285	34
Magaña (2012)	Tabasco	8 municipios de Tabasco	Tropical	ND	ND	495	ND
Mariaca-Méndez (2012)	Chiapas	Sureste Mexicano	Sub-humid/Tropical	ND	ND	418	ND
Pagaza-Calderón (2008)	Puebla	ND	Tempered	Totonaco/otomie/nahua/mestizo	53 homegardens	404	ND
Ruenes Morales y Montañez Escalante (2015)	Campeche	Hopelchén, Tenabo, Campeche, Champotón, Escárcega, Calakmul,	Tropical	ND	ND	174	ND
Ruenes Morales y Montañez Escalante (2015)	Quintana Roo	Othón P.B., J.M. Morelos, F.C. Puerto, Solidaridad	Tropical	ND	ND	310	ND
Ruenes Morales y Montañez Escalante (2015)	Yucatán	Tekax, Hocabá, Maní, Celestún, Telchac Puerto, Uman, Mérida, Abalá	Tropical	ND	ND	223	ND
WETLAND AGROFORESTRY SYSTEMS							
Local Reports							
Jiménez-Osornio y Gómez-Pompa (1990)	Distrito Federal	Tláhuac	Tempered	Mestizo	ND	146	ND
López-Ríos (1984)	Distrito Federal	Xochimilco	Tempered	Mestizo	ND	43	28
Nava (2007)	Tlaxcala	Ixtacuixtla de Mariano de Matamoros	Tempered	Mestizo	ND	56	34
Ochoa y Gonzáles (2009)	Campeche	Xicalanco	Tropical	Chontal	ND	37	ND
Osorio-Sánchez et al. (2004)	Tabasco	Nacajuca	Tropical	Chontal	ND	50	72
Venegas (1978)	Distrito Federal	Tláhuac	Tempered	Mestizo	ND	22	82
LONG FALLOW AGROFORESTRY							
Local Reports							
Berlín et al. (2001)	Chiapas	Región de los Altos de Chiapas	Sub-humid	Tzeltal/Tzotzil/Tojolabal	28 towns	199	ND
Blanco-Rosas (2006)	Veracruz	Soteapan	Tropical	Zoque popoluca	16 milpas	257	ND
Diemont et al. (2006)	Chiapas	Ocosingo	Tropical	Maya	6 farms	12	ND
Hellier et al. (1999)	Chiapas	Comitán	Sub-humid	Tojolabal/Mestizo	2 comunitys	60	22
LaRochelle (2003)	Chihuahua	Guachochi	Tempered	Raramurí	ND	59	34
Ochoa-Gaona et al. (2007)	Chiapas	Palenque/Ocosingo	Sub-humid	ND	39 plots	119	ND
Otero (2005)	Guerrero	Acapulco de Juárez	Sub-humid	Mestizo	ND	291	ND
Regional Reports							
Aguilar et al.	Guerrero	Zitlala/Chilapa/Ahuacuotzingo/Mártir de Cuilapan	Sub-humid	Nahua	ND	35	ND
AGROFOREST							
Local Reports							
Rosales Adame et al. (2014)	Jalisco y Nayarit	Villa Purificación Ruiz y Santiago Ixcuintla.	Sub-humid	ND	ND	69	ND
Baeza (2003)	Oaxaca	Santiago Nuyoo/Santa María Yucuite	Tempered	Ñuu Savi	12 plots	87	51
García Burgos et al. (2014)	Veracruz	Totutla	Sub-humid	ND	ND	13	ND
Gómez-Pompa et al. (2012)	Yucatán	Yaxcabá, Dzoncauich	Tropical	Maya	5 peet kotoob	48	ND
Ibarra et al. (2001)	Tabasco	Comacalco y Paraíso	Tropical	ND	ND	84	ND
Lucio-Palacio et al. (2015)	Chiapas	Tapachula	Tropical	ND	ND	76	ND
Martínez López et al. (2011)	Tabasco	Cunduacán	Tropical	ND	ND	25	ND
Muñoz et al. (2006)	Tabasco	Comalcalco	Tropical	ND	1 plot	16	ND
Peeters et al. (2003)	Chiapas	Jitolol de Zaragoza Plan de Paredón	Tropical	Zoque	ND	46	56
Ramírez López (2012)	Chiapas	Chenalhó	Tropical	Tsotsil	ND	138	ND
Ramírez-Meneses et al. (2013)	Tabasco	Cárdenas	Tropical	ND	ND	44	ND
Roa-Romero (2009)	Chiapas	Huehuetaán/Tapachula/Tuxtla Chico	Sub-humid	ND	28 plots	46	48
Salgado-Mora (2007)	Chiapas	Tuzántan/Huehuetán/Tapachula/Tuxtla Chico	Sub-humid	ND	80 plots	47	48
Sánchez-Gutiérrez (2012)	Tabasco	Cárdenas	Sub-humid	ND	20 plots	67	ND
Saucedo García et al. (2014)	Veracruz	Coatepec/Huatusco	Sub-humid	ND	ND	24	ND
Soto Pinto (2000)	Chiapas	Chilón	Tropical	Tzeltal	ND	61	41
Tlapaya y Gallina (2010)	Veracruz	Teocelo, Coatepec y Huastuco	Sub-humid	ND	ND	16	75
Ventura-Aquino (2008)	Oaxaca	San Agustin Loxica	Tempered	Zapoteco	ND	49	ND
Villavicencio y Valdez (2003)	Veracruz	Amatlán de los reyes	Tropical	Mestizo	34 plots	81	ND
Regional Reports							
Espejo Serna et al. (2005)	Sinaloa, Durango, Nayarit, Jalisco, Guerrero, Oaxaca, Chiapas, Tabasco, Veracruz, Puebla, Hidalgo, San Luis Potosí, Querétaro,	ND	Arid, sub.humid, tempered and tropical	ND	ND	213	ND
Martínez et al. (2007)	Puebla	Sierra Norte de Puebla	Tempered	Totonaco/Tepehua/Nahua/Otomie/Mestizo	ND	319	48
TERRACES AND SEMI-TERRACES AGROFORESTRY							
Local Reports							
Altieri y Trujillo (1987)	Tlaxcala	Tlaxcala	Tempered/Sub-humid	Mestizo	ND	22	14
Miranda et al. (2003)	Tlaxcala	Españita	Tempered/Sub-humid	Mestizo	ND	153	ND
Patrick (1977)	Tlaxcala	Miguel Hidalgo y Costilla	Tempered/Sub-humid	Mestizo	ND	3	ND
Pérez Sánchez (2012)	Tlaxcala	Ixtacuixtla	Tempered/Sub-humid	Mestizo	ND	25	24
ARID AND SEMIARID AGROFORESTRY							
Local Reports							
Blanckaert et al. (2007)	Oaxaca	Teotitlán de Flores Magón	Semi-arid	Mestizos y pocos Mazatecos	ND	43	7
Campos-Salas (2015)	Puebla	Atexcal	Semi-arid/Tempered	Mestizos	ND	69	ND
Hoogesteger et al. (2016)	Guanajuato	Xichú	Semi-arid/Tempered	Mestizos	9 plots	72	24
Moreno-Calles et al. (2012)	Oaxaca	Caltepec	Semi-arid	Mestizos	9 plots	122	16
Nabham et al. 1982	Sonora	Puerto Peñasco	Arid	Mestizos	38 homegardens	81	53
Stienen (1990)	Nuevo León, Tamaulipas y Coahuila	Linares	Semi-arid	Mestizos	ND	28	75
Regional Reports							
Moreno Calles et al. (2010)	Puebla/Oaxcaca	Caltepec/Zapotitlán Salinas/Santa María Tecomavaca	Semi-arid	Mestizos	6 plots	134	18
Nabham et al. (2010)	Baja California	Varios	Mediterranean/Arid	Mestizos	ND	71	ND
Vallejo Ramos (2015)	Puebla	Coyomeapan, San José Miahuatlán, San Pedro Ixcatlán, Concepción Pápalo, San Juan Bautista Cuicatlán, Zapotitlán	Semi-arid/Tempered	Náhuatl, Ixcatecos, Cuicatecos	15 plots	66	ND
AGROSILVOPASTORIL SYSTEMS							
Bautista (2009); Bautista-Tolentino et al. (2011)	Veracruz	Paso de ovejas	Warm/Sub-humid	ND	26 plots	14	ND
Jiménez-Ferrer et al.(2007)	Chiapas	Trinitaria	Tempered	ND	ND	13	ND
Ramírez-Marcial et al. (2012)	Chiapas	Ocozocouautla de Espinosa	Warm/Sub-humid	Zoque	5 Farms	59 in ASPS and SPS	ND
Vargas-López (2003)	Puebla	Cuautinchan/Tecali/Tzicatlacoyan/Puebla	Semi-arid/Tempered	ND	7 Farms	5	ND

## Appendix 2

**Table 3:** Reports by agroforestry system type in Mexico with animal species inventory

Autor	State	Species by report
HOMEGARDENS		
Álvarez Lugo (1997)	Veracruz	5
Cahuich Campos (2012)	Campeche	14
Chi (2012)	Campeche	8
Charblé-Santos et al. (2012	Yucatán	12
Cruz Bojórquez (2012)	Yucatán	1
Domínguez Santos et al. (2012)	Yucatán	57
Granados Sánchez et al. (1999)	Quintana Roo	9
Hernández Soto (2009)	Puebla	8
Mariaca Méndez 2012	Yucatán	47
Montañez Escalante et al. 2012	Yucatán	37
Neulinger et al. 2012	Campeche	12
Zaragoza et al. 2011	Chiapas	7
WETLAND AGROFORESTRY SYSTEMS		
Cahero 1997	Tabasco	9
Ochoa y González -Jácome (2009)	Campeche	35
Osorio et al. (2004)	Tabasco	28
Mariaca (1999)	Tabasco	6
Brown (1999)	Tabasco	8
Chávez (1999)	Tabasco	20
Pineda et al. (1999)	Tabasco	7
Pérez-Sánchez (2008)	Tabasco	14
LONG FALLOW AGROFORESTRY		
Blanco Rosas (2006)	Veracruz	18
Hellier et al. (1999)	Chiapas	28
Flores Cruz (2011)	Oaxaca	9
AGROFOREST		
Aragón y López-Paniagua (2015)	Puebla	99
De Haro (2006)	Veracruz	107
Gallina et al. (1996)	Veracruz	24
Ibarra et al. (2001)	Tabasco	84
Greenberg et al. (2000)	Tabasco	81
González-Ortega et al. (2011)	Chiapas	13
Cruz-Parra (2012)	Chiapas	21
Marcíp-Rios y Muñoz-Alonso (2008)	Chiapas	16
Mendoza-Sáenz (2012)	Chiapas	25
Brito-Ríos (2015)	Jalisco	39
Mérida-Rivas (2010)	Chiapas	27
Cruz-Lara et al. (2004)	Chiapas	43
Escamilla (2008)	Veracruz	3
De la Mora et al. (2008)	Chiapas	2
Philpott (2005)	Chiapas	6
Tlapaya y Gallina (2010)	Veracruz	18
Murieta-Galindo et al. (2013)	Veracruz	19
ARID AND SEMIARID AGROFORESTRY		
Zuria y Gates (2013)	Guanajuato (el Bajío)	61
Nabhan et al. (1982)	Sonora	43
Ortíz et al. (2010)	Puebla/Oaxaca	6

## Abbreviations

AFS: Agroforestry systems
CONEVAL: Consejo Nacional de Evaluación de la Política de Desarrollo Social
FAO: Food and Agriculture Organization of the United Nations
INEGI: Instituto Nacional de Estadística y Geografía
